# TBK1: a new player in ALS linking autophagy and neuroinflammation

**DOI:** 10.1186/s13041-017-0287-x

**Published:** 2017-02-02

**Authors:** James A. Oakes, Maria C. Davies, Mark O. Collins

**Affiliations:** 1 School of Medicine, University of Sheffield, Sheffield, UK; 2 Department of Biomedical Science, University of Sheffield, Firth Court, Western Bank, Sheffield, S10 2TN UK

**Keywords:** TBK1, Amyotrophic lateral sclerosis, ALS, Motor neuron disease, Autophagy, Mitophagy, Neuroinflammation, Signaling, Frontotemporal dementia, FTD

## Abstract

Amyotrophic lateral sclerosis (ALS) is an adult-onset neurodegenerative disorder affecting motor neurons, resulting in progressive muscle weakness and death by respiratory failure. Protein and RNA aggregates are a hallmark of ALS pathology and are thought to contribute to ALS by impairing axonal transport. Mutations in several genes known to contribute to ALS result in deposition of their protein products as aggregates; these include *TARDBP*, *C9ORF72,* and *SOD1*. In motor neurons, this can disrupt transport of mitochondria to areas of metabolic need, resulting in damage to cells and can elicit a neuroinflammatory response leading to further neuronal damage. Recently, eight independent human genetics studies have uncovered a link between TANK-binding kinase 1 (*TBK1*) mutations and ALS. TBK1 belongs to the IKK-kinase family of kinases that are involved in innate immunity signaling pathways; specifically, TBK1 is an inducer of type-1 interferons. TBK1 also has a major role in autophagy and mitophagy, chiefly the phosphorylation of autophagy adaptors. Several other ALS genes are also involved in autophagy, including p62 and OPTN. TBK1 is required for efficient cargo recruitment in autophagy; mutations in TBK1 may result in impaired autophagy and contribute to the accumulation of protein aggregates and ALS pathology. In this review, we focus on the role of TBK1 in autophagy and the contributions of this process to the pathophysiology of ALS.

**Electronic supplementary material:**

The online version of this article (doi:10.1186/s13041-017-0287-x) contains supplementary material, which is available to authorized users.

## Introduction

ALS is a multifactorial disorder with diverse genetic and environmental components [1]. The median incidence rate of ALS is approximately 2/100000 [2], of which 5–10% are familial (fALS). The mean age of onset of ALS is between 50 and 65 years with death occurring on average 2–3 years post-onset, due to respiratory failure [3]. Riluzole is currently the only drug available for treatment of ALS [4]; its modest therapeutic value highlights the imminent need for novel ALS treatments. Environmental factors linked to ALS include smoking and exposure to chemicals metals and radiation [5]. These factors are thought to contribute to ALS by a number of mechanisms; an important common factor is increased oxidative stress in neurons. Other mechanisms include damage to mitochondria, neurotoxicity and increased glutamate excitotoxicity by heavy metals (particularly lead) and cigarette smoke [3]. Mutations in more than 40 genes are thought to contribute to the disease. Amongst the most prevalent causes are *SOD1, C9ORF72, TARDBP, FUS*, *OPTN* and the most recently identified ALS gene TANK-binding kinase 1 (*TBK1)* [1, 6, 7].

### *TBK1* as an ALS gene

Genetic alterations in *TBK1* (also known as *NAK* or *T2K*) were initially associated with diseases with known neuroinflammatory components, including two forms of glaucoma: primary open angle glaucoma and normal tension glaucoma. Increased *TBK1* copy number resulting in a gain of function is recognized as a rare cause of these diseases [8, 9]. Heterozygous loss of function TBK1 mutations are also associated with herpes simplex encephalitis in childhood and it has been suggested that this is due to decreased activity in TLR3 mediated immunity [10].

*TBK1* was first identified as an ALS gene by two independent studies. Cirulli et al*.* [6] performed exome sequencing on 2869 ALS patients and 6405 controls of genetically European ethnicity and analyzed variants using a number of inheritance models. This study confirmed several previously identified ALS genes and identified *TBK1* as a novel ALS gene [6]. Freischmidt et al*.* [7] performed exome sequencing and a targeted mutation screen using high resolution melting curve analysis which identified *TBK1* as an ALS gene in a Swedish population. Subsequently, a study of Australian fALS patients identified a novel TBK1 mutation in a family of Chinese origin, the first TBK1 mutation found in an Asian ALS patient [11]. This study did not find any TBK1 mutations in patients of European ancestry and concluded that TBK1 mutations are rare in Australian fALS patients [11]. More recently, TBK1 mutations have been found to be a rare cause of ALS in Taiwanese and Chinese populations [12, 13], as well as in Sardinian ALS patients [14]. Frontotemporal dementia (FTD) is a neurodegenerative disorder closely linked to ALS and many patients present with both conditions. In a study of ALS and FTD patients in a French population, TBK1 mutations occurred more frequently in patients with FTD-ALS comorbidity (10.8%) than in those with ALS alone (0.5%) [15–17]. Several other studies have identified TBK1 mutations to be a major cause of FTD either concurrent with or without ALS [12, 14].

**Fig. 1:**
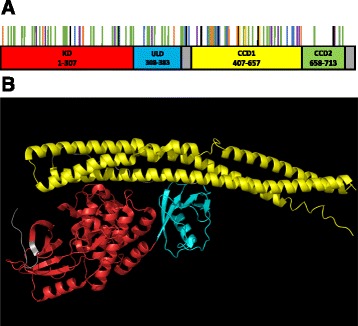
**a** Primary structure of TBK1. TBK1 is 729 amino acids in length and comprises four main domains: a kinase domain (KD), ubiquitin-like domain (ULD) and two coiled-coil domains (CCD1 and CCD2). Locations of TBK1 mutations associated with ALS and ALS-FTD are indicated by *vertical lines*: *Black* – frame shift mutations, *orange* – nonsense mutations, *green* – Missense mutations, *blue* – in-frame deletions, *purple* – splice mutations and *yellow* - insertion mutations. **b** Tertiary structure of TBK1. The region highlighted in red represents the kinase domain, the turquoise region represents the ubiquitin-like domain and the *yellow region* represents coiled-coil domain 1. Coiled-coil domain 2 is not shown in this model. Diagram produced in PyMOL using crystal structure data published by Larabi et al. 2013 [40] accessed via protein databank (accession number: 4IWO). (RCSB Protein Data Bank, 2016)

These human genetic studies identified nonsense, frameshift, missense and deletion mutations in both sporadic and familial ALS cases (and ALS-FTD/FTD) dispersed throughout the TBK1 protein sequence (Fig. 1a). Nonsense and frameshift mutations cause major disruption to TBK1 and may decrease its expression at both the mRNA and protein level [7, 17], implying that TBK1 haploinsufficiency contributes to the development of ALS in these cases. However, the contribution of missense mutations and single amino acid deletions are more subtle, as they may or may not confer either loss of function or reduction in function. These variants are also less likely to cause a decrease in expression suggesting haploinsufficiency is not to blame in these cases. Elucidation of the functional effects of these variants will contribute to our understanding of ALS pathogenesis as well as TBK1 function as discussed below.

### Molecular pathology of ALS

ALS involves the premature loss of upper and lower motor neurons, with degeneration of these neurons in the spinal cord, brainstem and motor cortex [3]. This leads to weakening and atrophy of muscles and eventually paralysis [1, 3]. At the molecular level, numerous factors affect ALS pathology including RNA and protein aggregates, mitochondrial dysfunction and neuroinflammation.

#### Protein and RNA aggregates

The presence of protein and RNA aggregates in the cytoplasm of motor neurons is the primary hallmark of ALS. The most common protein inclusion in ALS is TDP-43 (encoded by *TARDBP*). *TARDBP* mutations are a cause of both fALS and sporadic ALS (sALS); however, mutations are not a requirement for formation of aggregates, which may be formed from wild-type proteins. TDP-43 is an RNA-binding protein normally found in the nucleus where it has roles in gene expression and RNA processing. However, cytoplasmic aggregates of TDP-43 are frequently observed in ALS and these are often ubiquitinated [1].

C9ORF72, which has roles in stress granule formation, microglial function and autophagy [18–20], contains a hexanucleotide GGGGCC repeat in its first intron; expansion of this repeat is the most frequent cause of fALS [21]. In healthy individuals, the repeat is <30 copies in length, however, in ALS patients the number of repeats may be expanded to hundreds of copies. *C9ORF72* expanded repeats result in three types of inclusion: intranuclear RNA foci, cytoplasmic C9ORF72 aggregates, (which may colocalize with p62 and TDP-43), and dipeptide repeat proteins produced by RAN-translation of the expanded region [1, 22]. These abnormal protein aggregates are thought to be the mechanism by which *C9ORF72* expanded repeats contribute to ALS. *C9ORF72* transcription in heterozygous 311005021Rik (*C9ORF72* mouse ortholog) knock-in C57BL/6 mice is highest in neurons of the hippocampus, cerebellum, cortex, brainstem nuclei and striatum, all of which are known to degenerate in ALS and FTD [23]. This suggests that *C9ORF72* repeat expansion related pathology would be most potent in these regions, providing some insight into the cell-specificity of neuronal degeneration in *C9ORF72*-ALS.

*SOD1* was the first ALS gene identified and it is widely studied due to the availability of the SOD1-G93A mouse model of ALS [24]. Mutations in *SOD1* produce an unstable protein which is deposited in the cytoplasm; oligomerisation of unstable SOD1 leads to aggregate formation [25]. FUS is another RNA binding protein in which mutations can result in the formation of cytoplasmic aggregates. It is a component of stress granules and may form p62 and TDP-43 positive aggregates [1]. TDP-43 positive protein inclusions, as well as *C9ORF72* repeat expansion pathologies, are also involved in FTD and ALS-FTD.

The mechanism by which these inclusions contribute to ALS and FTD pathogenesis is as yet undefined, though they are thought to impair axonal transport [3]. It is also unclear as to whether the inclusions themselves are cytotoxic or if they are secondary to another primary pathology [1, 22]. It is postulated that impaired autophagy could be a contributing factor to the accumulation of cytoplasmic aggregates [26]. TBK1 has important roles in autophagy [27] and it seems likely that it is by this mechanism that *TBK1* mutations contribute to ALS.

#### Mitochondrial dysfunction

Mitochondria have important roles in cellular respiration, calcium buffering and apoptosis. Neurons are particularly sensitive to mitochondrial dysfunction given their high metabolic rate [28] and the presence of abnormal or dysfunctional mitochondria in neurons is thought to be a contributing factor in ALS. The presence of mutant SOD1 in the cytoplasm of motor neurons plays a major role in mitochondrial dysfunction resulting in impaired ATP production, impaired calcium buffering and early apoptosis of neuronal cells [29–31]. Calcium is critical to the correct functioning of motor neurons as it has major roles in metabolism, development, and synaptic transmission. Early apoptosis is brought about by the interaction of SOD1 and mitochondrial apoptotic machinery, this process directly contributes to motor neuron degeneration [32].

Transport of substances along the length of motor neurons is crucial for normal function and disruption of axonal transport of mitochondria has been observed in both ALS patients and animal models [33, 34]. Mitochondria are transported along motor neurons to areas of greatest metabolic need and calcium regulation; disruption of this process leads to reduced ATP availability and dysregulation of calcium levels resulting in neuron damage. Disruption of axonal transport also potentiates the accumulation of protein and RNA aggregates discussed above, this leads to further impairment of axonal transport resulting in motor neuron degeneration [3]. Damage to motor neurons as a result of protein aggregate accumulation and mitochondrial dysfunction leads to secondary non-neuronal cell causes of motor neuron degeneration such as neuroinflammation [1].

#### Neuroinflammation

Glial cells are the principle innate immune cell of the CNS and pathology associated with these cells is referred to as neuroinflammation [35], a hallmark of ALS. Glial cells primarily express the Toll-like receptors TLR3 and TLR4 [36]; ligand binding results in activation and migration of glial cells towards sites of damage where they dispose of damaged cells through phagocytosis. A by-product of this process is the production of neurotoxic molecules such as pro-inflammatory cytokines and reactive oxygen species. These molecules may cause further neuronal damage leading to further glial cell activation resulting in a positive feedback loop of neuroinflammation [35]. TBK1 is involved in the innate immune response by regulating the production of IFNα and IFNβ. Ligand binding of TLR3 or TLR4 results in recruitment of adaptor proteins TRIF and TRAM. TRIF interacts with TRAF3 resulting in activation of TBK1/IKKi hetero- or homo-dimers which in turn phosphorylate IRF-3 and IRF-7 allowing the formation of homodimers. IRF-3/IRF-7 homodimers are transported to the nucleus where they act as transcription factors for IFNα and IFNβ [37]. TBK1 is also involved in TLR independent antiviral signaling involving intracellular RIG-I-like receptors (RLRs). Ligand binding of RLRs leads to TBK1 activation through a complex pathway involving the outer mitochondrial membrane (OMM) protein MAVS and the adaptor protein STING [38].

T cells have been observed in spinal cord lesions of ALS patients and are thought to play a role in the regulation of neuroinflammation. CD4+ cells stabilize microglial activation, decrease pro-inflammatory cytokines and increase growth factor IGF-1 suggesting that T cells play a protective role in ALS [35]. Migration of T cells from the lymph nodes is impaired in *TBK1* knockout mice [39], potentially resulting in decreased T cell number in the CNS. This may increase the damage caused by neuroinflammation by removing the protective regulation by T cells.

### TBK1 structure and function

#### Protein structure and regulation

TBK1 contains four domains: a serine/threonine kinase domain (KD) (residues 1–307) located at its N-terminal, a ubiquitin-like domain (ULD) (residues 308–383) and two coiled-coil domains, coiled-coil domain 1 (CCD1) (residues 407–657) and coiled-coil domain 2 (CCD2) (residues 658–713) http://www.uniprot.org/uniprot/Q9UHD2 [40]. (Fig. 1). CCD1 is also referred to as a scaffold dimerization domain (SDD) [7, 40, 41]. TBK1 may form a homodimer or a heterodimer with IKKi; formation of a homodimer is primarily mediated by interactions between the two CCD1 domains however, the KDs and ULDs also interact with the adjacent molecule. The interacting residues forming the dimer are conserved and are required for activation, a process that requires TBK1 dimerisation [42].

The KD is comprised of two lobes termed N-terminal and C-terminal lobes, with the active site situated between these two lobes. The KD also contains an activation loop (Leu164-Gly199) which includes Ser172, phosphorylation of which brings about TBK1 activation. In the inactive form, the activation loop protrudes away from the KD towards the C-lobe of the KD of the other TBK1 protein comprising the dimer. A conserved residue, Glu55, is also displaced from the active site. It is speculated that TBK1 dimers are able to autophosphorylate through the interaction of adjacent KDs and activation loops. Phosphorylation of Ser172 results in a conformational change of the activation loop. The activation loop retracts towards and interacts with its own KD allowing substrate binding. The conserved Glu55 residue is also rotated into a position where it can form a salt bridge with Lys38. Other than this, conformational changes as a result of phosphorylation are limited [40, 43].

Poly-ubiquitination of Lys30 and Lys401 is a requirement for activation of TBK1 and a multistep mechanism of TBK1 activation beginning with poly-ubiquitination of Lys30 and Lys401 followed by phosphorylation of Ser172 has been suggested [42]. This results in a conformational change of the active site to allow substrate binding. Along with the KD, the ULD is also important for the kinase activity of TBK1; deletion of the ULD results in a loss of kinase activity [44]. Sequence alignment of the TBK1 ULD with similar human ULDs and ULDs of TBK1 from other species has identified many structurally important conserved residues. Three residues, Leu316, Ile353, and Val382, are suspected to be involved in protein-protein interactions [41]. These residues form a hydrophobic patch homologous to the hydrophobic patch of ubiquitin (Leu316, Ile44, and Val70) [41]. Ubiquitin interacts with its binding partners via this hydrophobic patch, indicating that TBK1 may also interact with proteins via this structure. The hydrophobic patch is also thought to be involved in interactions between the TBK1 ULD and CCD1. Comparison of the surface charge of various regions of ULDs of IKK-family kinases reveals that it differs between family members and these differences may determine substrate specificity [41]. Mutations around the hydrophobic patch have been demonstrated to prevent activation of downstream molecules of TBK1 [41].

TBK1 is regulated by adaptor proteins that control its localization, activation and participation in different signaling pathways [45, 46]. TBK1 possesses the ability to robustly autophosphorylate, and therefore requires strict regulation. This, alongside the fact that TBK1 plays roles in many pathways including induction of interferons and autophagy, means that the subcellular localization of TBK1 may contribute to its regulation as well as its signaling specificity [47]. NAP1, TANK, and Sintbad are adaptor proteins that bind to the CCD2 domain of TBK1. These adaptor proteins bind in a mutually exclusive manner and the differing subcellular localization of these adaptors may determine the pathway in which TBK1 will participate [46]. This is supported by findings that TBK1 activated in autophagy does not result in activation of its downstream targets in the innate immunity signaling, this suggests that there is limited crosstalk between the different TBK1 pathways [48]. NAP1 and Sintbad are localized diffusely throughout the cytoplasm whereas TANK is punctate in the perinuclear region, this has led to suggestions that binding of TANK results in the induction of IFNα and IFNβ whereas binding of NAP1 or Sintbad is more important for autophagy [49]. A network of 30 proteins interacting with one or more of these adaptor proteins and/or TBK1/IKKi has been established [46]. This all suggests that adaptor binding to TBK1 plays a key role in its activation and function within the cell.

#### TBK1 mutations

We collated 92 TBK1 mutations identified in patients with ALS, ALS-FTD or FTD from eight human genetics studies (Table 1, Additional file 1: Table S1 and Fig. 1). 88 of these mutations were identified in ALS patients (ALS and ALS with FTD) of which 27 are potential loss of function variants (nonsense, splice site and frameshift mutations) and 16 are in-frame insertions/deletions. Freischmidt et al. identified 8 heterozygous loss of function variants, of which 7 resulted in the loss of expression of TBK1 and therefore were reported as causative via haploinsufficiency [7]. The loss of function variant that was expressed (p.690-713del) contained a deletion in the CCD2 domain that prevented binding of OPTN, indicating that this may be sufficient to cause ALS/FTD [7].

**Table 1:** Breakdown of TBK1 mutations by disease type and protein domains

	Domain			
Disease	Kinase domain	Ubiquitin-like domain	CCD1	CCD2
ALS	32 (84%)	11 (100%)	29 (71%)	2 (25%)
ALS-FTD	2 (5%)	0 (0%)	9 (22%)	4 (50%)
FTD	4 (11%)	0 (0%)	3 (7%)	2 (25%)
Total no. cases	38	11	41	8

**Fig. 2:**
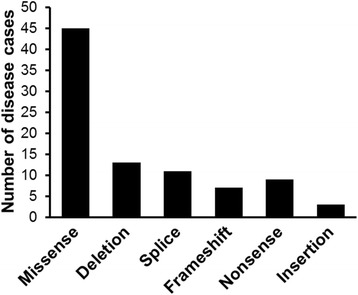
Frequency of different types of ALS and FTD-associated TBK1 mutations. Summary of data compiled in Additional file 1: Table S1

The majority of ALS-associated TBK1 variants (45) identified to date are missense mutations (Fig. 2, Additional file 1: Table S1) of unknown pathogenicity. The functional relevance of these variants is less obvious than nonsense or frameshift mutations, which lead to loss of expression of TBK1. Missense mutations may be pathogenic if the site is crucial to the function or stability of the protein. Mutations in the KD may affect phosphorylation of substrates whereas mutations within the ULD may affect recruitment to ubiquitinated proteins and organelles. CCD1 is important for TBK1 dimerisation and mutations here could affect this process, which is required for TBK1 activation [42]. CCD2 domain mutations could interfere with adaptor binding and TBK1 activation [46].

Mutations in the KD and CCD1 account for a greater proportion of disease cases than mutations in the ULD or CCD2 (Table 1, Additional file 1: Table S1) but when domain size is taken into account, mutations are evenly distributed across all domains of TBK1 (Fig. 1). KD, ULD and CCD1 mutations occur more frequently in ALS cases whereas CCD2 mutations seem more likely to result in ALS-FTD or FTD. However, larger numbers of patients with TBK1 mutations are needed to confirm any potential domain-specific associations. Using tools to predict the functional effects of mutations, the largest proportion of missence mutations that are probably damaging, occur in the kinase domain (Additional file 1: Table S1). However, biochemical analysis of variants located in each domain of TBK1 revealed functional deficits in TBK1 in four out of five cases, indicating that many missence mutations have the potential to be pathogenic [7]. Determining which TBK1 missence mutations cause selective loss of function of TBK1 could shed light on the functions of TBK1 most relevant to disease.

### Autophagy and the role of TBK1

#### Autophagy

Autophagy is a process by which ubiquitinated proteins and damaged organelles are degraded and recycled. Abnormal protein aggregates are a hallmark of ALS pathology, in addition to this, mutations in several genes involved autophagy have been associated with ALS including *SQSTM1* (encodes p62), *SOD1, OPTN*, *VCP, UBQLN2* and most recently *TBK1*. This suggests that disruption of autophagy is important in ALS pathophysiology [1, 50].

**Fig. 3:**
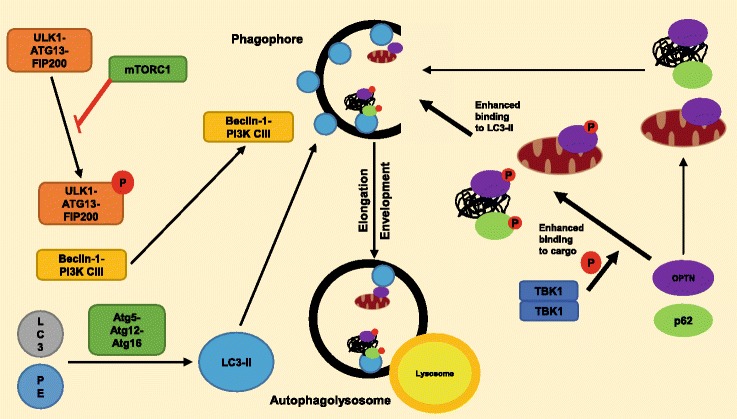
Autophagolysosome formation and maturation. Autophagy involves the formation of an isolation membrane. This membrane undergoes elongation, forming a phagophore and a portion of the cytoplasm is separated to form an autophagosome. This involves the ULK1-ATG13-FIP200, Beclin1-PI3K CIII and Atg5-Atg12-Atg16 complexes The autophagosome fuses with a lysosome to form an autophagolysosome, allowing digestive enzymes to degrade any material in the vesicle. TBK1 binds and phosphorylates autophagy receptors OPTN and p62; they bind to ubiquitin residues on target cargo and to LC3-II. This enhances the ability of the receptors to bind ubiquitinated residues on target cargo and LC3-II. This all allows ubiquitinated cargo to be recruited to the phagophore for degradation; the receptors act as adaptors that link cargo to autophagic machinery

Autophagy begins with the formation of an immature membrane structure called a phagophore in response to signaling initiated by phosphorylation of the ULK1-ATG13-FIP200 complex [51]. This is normally inhibited by mTORC1, however, the action of mTORC1 can, in turn, be inhibited by AMPK [26]. The activated ULK1-ATG13-FIP200 complex triggers movement of another complex containing beclin1 and PI3K CIII towards a phagophore. This complex mediates elongation of the phagophore membrane and envelopment of proteins or organelles marked for degradation, resulting in the formation of a double-membrane bound autophagosome. The process of autophagosome formation is also mediated by two interlinked control systems, the Atg5-Atg12 conjugation system [52] and the microtubule-associated protein 1A/1B LC3 conjugation system [53]. The Atg5-Atg12 conjugation system results in the formation of a complex of Atg5-Atg12-Atg16. This complex allows conjugation of LC3 with phosphatidyl-ethanolamine to produce LC3-II [50], which then binds to the surface of a phagophore where it has roles in elongation and cargo recruitment [26]. The autophagosome is transported along a microtubule to a lysosome-rich area. The autophagosome fuses with a lysosome forming an autophagolysosome, the contents of which are digested [50] (Fig. 3).

Autophagy adaptors enhance disposal of cargo, such as ubiquitinated proteins and damaged mitochondria, by linking them with autophagosome-associated proteins such as LC3-II [50]. Many of the ALS-associated inclusions described previously contain p62 and ubiquitin. Ubiquitination of proteins marks them for degradation; autophagy adaptors recruit ubiquitinated proteins to the phagophore by linking ubiquitin and LC3-II. p62 and OPTN are autophagy adaptors and mutations in these proteins result in impaired autophagy and are recognized causes of ALS [54, 55]. Importantly, p62, OPTN, and another autophagy adaptor NDP52 are all regulated by phosphorylation by TBK1 [48].

The presence of abnormal protein inclusions in the brain is a common feature of ALS and neurons can respond to this accumulation by upregulating autophagy [56]; if autophagy is disrupted, this may contribute to neurodegeneration and the pathology of ALS. This hypothesis is supported by the loss of autophagy in the CNS of Atg7^−/−^ and Atg5^−/−^ mice (two genes essential for autophagy), resulting in neurodegenerative disease [57, 58]. Loss of neurons, increased apoptosis and increased protein aggregation were evident when autophagy was decreased [57, 58]. The fact that several ALS genes are involved in autophagy and the knowledge that formation of protein aggregates contribute to ALS pathogenesis, suggests that disruption of autophagy is of importance to the development of ALS. The various roles of TBK1 in autophagy, especially adaptor phosphorylation, suggests that this may also be an important process in the contribution of TBK1 mutations to ALS.

TBK1 also plays a role in innate immunity, chiefly the induction of type-I interferons, alongside its aforementioned role in autophagy. These roles seem to be largely distinct and only limited crosstalk is evident. Interferons, induced by TBK1, lead to the transcription of interferon-inducible genes, amongst the targets of type-I interferons is *ISG15*, which has been linked to autophagy [59, 60]. ISG15 interacts with p62 and HDAC6, a protein with roles in cargo aggregation and autophagolysosome formation [61]. Overexpression of ISG15 results in enhanced aggregate formation and autophagy [60]. ISG15 can also suppress autophagy by binding beclin1 and PI3K CIII [59]; proteins important in phagophore elongation [26]. Loss of TBK1 decreases ISG15 and therefore removes its suppression of autophagy. In addition to this, enhanced aggregate formation was only observed with overexpression of ISG15 [60]. These factors suggest that ISG15 related mechanisms are probably not important in the contribution of mutant *TBK1* to ALS; however, this highlights an interesting link between two TBK1 pathways.

#### Autophagy, TBK1 and ALS pathogenesis

TBK1 plays key roles in autophagy, including the phosphorylation of a number of autophagy adaptors including p62, OPTN and NDP52 [48], enhancing their ability to link LC3-II and ubiquitinated cargo (Fig. 3) [26]. Phosphorylation of OPTN and p62 involves interaction with the CCD2 domain of TBK1 [15]. Several *TBK1* mutations identified in ALS patients cause protein truncation resulting in loss of CCD2 [7], decreasing the ability of TBK1 to phosphorylate these molecules. In addition to this, some *TBK1* mutations result in decreased mRNA and protein levels [7, 17], which may decrease activation of autophagy adaptors, resulting in decreased autophagy and accumulation of protein aggregates in motor neurons.

TBK1 has also been implicated in autophagosome maturation. In TBK1 knockdown cells, autophagosome formation was not disrupted whereas maturation of autophagosomes into autophagolysosomes was inhibited [27]. Rab8b is an upstream regulator of TBK1 and they both colocalize with LC3 on autophagosomes; this interaction is proposed as a mechanism by which TBK1 is involved in autophagosome maturation [27]. Maturation of autophagosomes involves transport to a lysosome-rich area via microtubules, this is dependent on the action of the motor protein dynein [62]. TBK1 regulates microtubule dynamics in mitosis and the cytoplasmic levels of dynein [63]. Disruption of microtubule transport due to loss of TBK1 could contribute to ALS by the impaired maturation of autophagosomes into autophagolysosomes.

Several studies have attempted to characterize neuropathological features associated with TBK1-ALS and FTD, though overall the number of individuals examined is still low. An ALS patient carrying a TBK1 mutation has been found with both TDP-43 positive and p62 positive inclusions in motor neurons as well as TDP-43 inclusions in the cortex [64]. An FTD patient from the same study was found to have TDP-43 inclusions in various brain regions as well as cytoplasmic p62 and ubiquitin-positive inclusions in glial cells [64]. Another study found TDP-43 positive inclusions in three out of five TBK1-FTD patients [17] and another found both TDP-43 and p62 positive inclusions in various brain regions of a TBK1-FTD/ALS patient [7]. These findings, particularly of p62 and ubiquitin-positive inclusions, provide further indications that TBK1 mutations may contribute to ALS through impaired autophagy.

#### Mitophagy, TBK1 and ALS pathogenesis

Recent studies have identified important roles for TBK1 and other ALS genes in autophagy of mitochondria, specifically known as mitophagy [48, 65]. In the PINK1-Parkin pathway, PINK1 is able to detect damaged mitochondria by crossing the outer mitochondrial membrane (OMM). If the mitochondrion is healthy, PINK1 passes through the OMM and is degraded on the inner mitochondrial membrane (IMM). However, if the mitochondrion is damaged PINK1 is retained on the OMM where it accumulates and phosphorylates ubiquitin chains on several OMM proteins resulting in binding of autophagy adaptors [48]. PINK1 concurrently recruits and phosphorylates Parkin, resulting in its activation. Parkin is an E3 ubiquitin ligase that constructs ubiquitin chains on OMM proteins, which are then phosphorylated by PINK1, allowing further adaptor binding [65]. TBK1 can then be activated by a mechanism dependent on Parkin, OPTN, NDP52 and OPTN-ubiquitin binding ability [48]. Activated TBK1 can phosphorylate NDP52, OPTN, and p62, greatly enhancing their ability to link ubiquitin and LC3-II [65]. Phosphorylation of OPTN enhances its ubiquitin binding ability and its role in TBK1 activation resulting in a positive feedback mechanism of TBK1 and OPTN activation [48, 65]. NDP52 and OPTN are able to bring about mitophagy by linking ubiquitinated OMM proteins and LC3 proteins on phagophores resulting in engulfment and digestion of mitochondria [26].

The role of p62 in mitophagy is controversial; several studies have reported that p62 is not essential for mitophagy but plays a role in the aggregation of ubiquitinated mitochondria [48, 65]. On the other hand, it has also been reported that activation of p62 is required for efficient mitophagy [66]. TBK1 is required for efficient recruitment of autophagy adaptors and efficient mitophagy, and TBK1 is essential for mitophagy via OPTN [48, 65]. Loss of TBK1 function would result in impaired mitophagy and accumulation of defective mitochondria, which may contribute to ALS by disrupting axonal transport that occurs in ALS [67].

Recent studies have added further weight to the argument that TBK1 mutations contribute to ALS through impaired autophagy/mitophagy. The E696K missense mutation identified in ALS patients [7, 17] occurs in the TBK1 CCD2 domain that interacts with adaptor proteins such as OPTN. This mutation disrupts two hydrogen bonds with H52 and K55 on the N-terminal domain of OPTN, which in turn disrupts the OPTN-TBK1 complex. Co-localization of TBK1 with OPTN is also reduced following the E696K mutation, with co-immunoprecipitation studies showing that the mutation almost completely abolishes the interaction between the two proteins [68]. Whilst wild-type TBK1 localizes to damaged mitochondria, the E696K mutant exhibited severely reduced colocalization with damaged mitochondria [69]. This suggests that this process is dependent on OPTN, due to the loss of TBK1-OPTN binding in the E696K mutant. This potentially provides evidence that defective mitophagy is a pathogenic mechanism in TBK1-ALS.

#### Tissue specificity and importance of age in TBK1-ALS

Autophagy can play one of two roles in the cell; to degrade proteins, regardless of cell stress, via continuous operation at low levels (basal autophagy) or to supply amino acids for cell survival during poor environmental conditions (adaptive autophagy) [70]. Basal autophagy in neuronal cells is crucial to their integrity. Most cells can dilute damaging agents through cell division, whereas the post-mitotic nature of neuronal cells means that they require autophagy to remove toxic proteins [71]. This implies a high level of basal autophagy in these cells, an idea supported by the high autophagic efficiency suggested by the rarity of autophagic vacuoles in healthy neurons [72]. Given the high level of autophagic activity required for neuronal maintenance, it is not surprising that these tissues are particularly susceptible to damage through disruption of autophagy.

TBK1 is normally expressed diffusely in the cytoplasm at moderate levels in all tissues but is expressed at a much higher level in neuronal cells of the cerebral cortex, hippocampus and lateral ventricle [73]. Moderate TBK1 levels are also seen in the glial cells of the cerebral cortex, Purkinje cells and granular layer cells in the cerebellum [73]. ALS is characterized by loss of spinal, cerebellar, hippocampal, brain stem and cortical motor neurons [23], as well as a loss of pyramidal neurons in the primary motor cortex. The fact that TBK1 expression is high in neurons of the CNS which are then lost during ALS, may suggest that loss of function TBK1 mutations may have a greater effect in these cells. TBK1 is also highly expressed in various other tissues, including the lungs, endocrine tissues and skin [73]. This begs the question as to why the major effects of ALS are only observed in nervous tissue. This may be explained by a threshold for development of pathology that may not be reached in these other tissues.

Advanced age appears to be an important factor in the development of ALS and this may be due to a number of factors. A reduction in the number of motor neurons is a normal part of aging and this can contribute to sarcopenia, the age-related loss of muscle mass [74]. In addition to this, the uptake of heavy metals into spinal cord neurons is also increased with advancing age [74]; exposure to heavy metals is a risk factor of ALS due to increased glutamate excitotoxicity [3]. Recovery of motor function is decreased in an aged ALS mouse model when compared to their younger counterparts [75]. Finally, the rate of autophagy declines with age leading to a reduction of the ability of cells to remove protein aggregates [76], or indeed damaged mitochondria which may be particularly detrimental to neurons. These factors may contribute to the tissue specificity of TBK1 involvement in ALS, regardless of its widespread expression. It is unlikely that defects in TBK1 related pathways suddenly appear at some point in the course of advancing age but instead the threshold required to cause disease is lowered to the point where these defects become pathological. In other words, age-related factors such as sarcopenia, heavy metal accumulation, and impaired neuronal recovery may facilitate the progression of ALS due to defective TBK1 signaling.

### Future outlook

TBK1 is involved in a variety of ALS-relevant pathways such as autophagy and neuroinflammation. TBK1 function, pathological findings in ALS and known pathogenic mechanisms of ALS point towards autophagy as the major contribution of TBK1 mutations to ALS. Defective autophagy may lead to the accumulation of protein aggregates, autophagosomes and damaged mitochondria in motor neurons. This may all result in impaired axonal transport of molecules and organelles, such as mitochondria, which are crucial for neuron function and survival. Given the varied and crucial roles of TBK1 in autophagy and mitophagy, it seems that these mechanisms may be paramount in the contribution of TBK1 to ALS pathogenesis. Impaired mitochondrial function and transport may result in neuronal damage by the mechanisms discussed above. Neuronal damage may trigger innate responses by cells surrounding neurons leading to neuroinflammation, another important mechanism in ALS pathogenesis. Further dissection of TBK1 signaling pathways in neurons will help further our understanding of the contribution of *TBK1* mutations to ALS. Given a large number of downstream targets of TBK1 that have been identified, relatively few have been investigated thoroughly. Studies comparing the pathological findings in different ALS genotypes may also aid our understanding of how different genes/pathways contribute to ALS.

## Additional file

Additional file 1: Dataset of TBK1 mutations identified in ALS/FTD patients compiled from the literature. (PDF 478 kb)

## Abbreviations

ALS: Amyotrophic lateral sclerosis
AMPK: AMP-activated protein kinase
Atg(5,12,13,16): Autophagy-related (5,12,13,16)
ATP: Adenosine triphosphate
C9ORF72: Chromosome 9 open reading frame 72
CCD(1,2): Coiled-coil domain
CNS: Central nervous system
fALS: Familial amyotrophic lateral sclerosis
FIP200: Focal adhesion kinase family interacting protein of 200 kD
FTD: Frontotemporal dementia
FUS: Fused in sarcoma
HDAC6: Histone deacetylase 6
IFN(α,β): Interferon
IFN: Interferon
IGF-1: Insulin-like growth factor 1
IKK(α,β,ε/i): IκB kinase
IMM: Inner mitochondrial membrane
IRF(3,7): Interferon regulatory factor
ISG15: Interferon-stimulated gene 15
IκB: Inhibitor of kappa B
KD: Kinase domain
LC3: Microtubule-associated protein 1A/1B-light chain 3
MAVS: Mitochondrial antiviral signalling protein
mTORC1: Mammalian transport of rapamycin complex 1
NAK: NFκB activating kinase
NAP1: Nucleosome assembly protein 1
NDP52: Nuclear dot protein 52 kDa
NFκB: Nuclear factor kappa-light-chain-enhancer of activated B cells
OMM: Outer mitochondrial membrane
OPTN: Optineurin
PI3K CIII: Phosphoinositide 3-kinase class III
PINK1: Phosphatase and tensin homolog (PTEN)-induced putative kinase 1.
RAN-translation: Repeat-associated non-ATG translation
RLR: Rig-I-like receptor
sALS: Sporadic amyotrophic lateral sclerosis
SDD: Scaffold dimerisation domain
SOD1: Superoxide dismutase 1
SQSTM1: Sequestosome-1
STING: Stimulator of interferon genes
T2K: TRAF2 kinase
TANK: TRAF family member-associated NF-kappa-B activator
TARDBP/TDP-43: Transactive response DNA binding protein 43
TBK1: TANK-binding kinase 1
TLR(3,4): Toll-like receptor
TRAF(2,3): TNF receptor-associated factor
TRAM: Translocating chain-associating membrane protein
TRIF: TIR-domain-containing adapter-inducing interferon-β
UBQLN2: Ubiquilin-2
ULD: Ubiquitin-like domain
ULK1: Unc-51 Like Autophagy Activating Kinase 1
VCP: Valosin-containing protein
