# Supplementary material for: TBK1: a new player in ALS linking autophagy and neuroinflammation
Source: Mol Brain. 2017 Feb 2;10:5. doi: 10.1186/s13041-017-0287-x (PMC5288885; doi:10.1186/s13041-017-0287-x)
Supplement: Additional file 1: — Dataset of TBK1 mutations identified in ALS/FTD patients compiled from the literature. (PDF 478 kb) [file 13041_2017_287_MOESM1_ESM.pdf]

Additional file 1: Table S1

Mutation	Type of Variant	Location	Disease	PolyPhen2	SNPs3D	SIFT	Age of onset	Survival Time	Refs
p.Q2X	Nonsense	KD	ALS, FTD				56	4	[6, 19, 78, 79]
p.T4A	Missense	KD	FTD	0.984	-1.84	0.00			[18, 78, 79]
p.L11S	Missense	KD	ALS	0.960	-1.2	0.02			[6, 78, 79]
p.N22H	Missense	KD	ALS	0.999	-0.17	0.03			[6, 78, 79]
p.N22D	Missense	KD	ALS	0.997	-0.52	0.13			[6, 78, 79]
p.R25H	Missense	KD	ALS	1.000	0.11	0.01			[6, 78, 79]
p.G26E	Missense	KD	ALS	1.000	-2.06	0.01			[6, 18, 78, 79]
p.I43V	Missense	KD	ALS	0.071	1.05	1.00	44		[15, 79]
p.R47H	Missense	KD	ALS	1.000	-2.39	0.00			[7, 78, 79]
p.L62P	Missense	KD	ALS	0.999	-3.11	0.00	47	2	[16]
p.T77WfsX4	Splice	KD	ALS				35, 58	5, 3	[7, 78, 79]
p.Y105C	Missense	KD	ALS, ALS-FTD	0.938	0.35	0.00			[6, 7, 78, 79]
p.R117X	Nonsense	KD	ALS				68	4	[6, 20, 78, 79]
p.V120Afs	Splice	KD	ALS						[6, 79]
p.N129D	Missense	KD	ALS	0.906	-2.23	0.06			[6, 7, 79]
p.V132E	Missense	KD	ALS	0.992	-3.51	0.00			[6, 78, 79]
p.R134H	Missense	KD	ALS	1.000	-3.08	0.00			[6, 78, 79]
p.R143C	Missense	KD	ALS	1.000	-0.29	0.00			[18, 78, 79]
p.S151C	Missense	KD	ALS	0.999	0.34	0.00			[6, 78, 79]
p.S151F	Missense	KD	ALS	0.997	-1.03	0.00			[6, 78, 79]
p.T156RfsX6	Deletion	KD	ALS-FTD						[6, 18, 78, 79]
p.D167del	Deletion	KD	ALS				60	1	[19, 78, 79]
p.180sp	Splice	KD	ALS						[6, 78]
p.Y185X	Nonsense	KD	ALS				47, 37, 41, 40	6, 3, 6, 3	[7, 78, 79]
p.G217R	Missense	KD	ALS	1.000	-3.42	0.00			[6, 78, 79]
p.R228H	Missense	KD	ALS	1.000	-2.05	0.00			[6, 78, 79]
p.I257T	Missense	KD	ALS	0.991	-1.84	0.01			[6, 78, 79]
p.R271L	Missense	KD	ALS	0.001	1.47	0.32	80	7	[19, 78, 79]
p.G272_T331del	Splice	KD	ALS						[6, 78, 79]
p.G272_T331del	Splice	KD	FTD						[19, 79]
p.L277V	Missense	KD	ALS	0.856	0.24	0.01			[6, 78, 79]
p.T278fs	Frameshift	KD	ALS						[6, 78]
p.P279Cfs	Deletion	KD	ALS						[6, 79]
p.K291E	Missense	KD	FTD	0.997	-1.8	0.01	60	4	[19, 78, 79]
p.G294D	Missense	KD	ALS	1.000	-0.09	0.01	46		[15, 79]
p.I305T	Missense	KD	ALS	0.770	-0.92	0.07			[6, 7, 78, 79]
p.L306I	Missense	KD	ALS-FTD	0.749	-1.21	0.02	70	2	[6, 7, 78, 79]

Mutation	Type of Variant	Location	Disease	PolyPhen2	SNPs3D	SIFT	Age of onset	Survival Time	Refs
p.R308Q	Missense	ULD	ALS	0.999	-0.01	0.07	38	4	[7, 78, 79]
p.T320QfsX40	Deletion	ULD	ALS				60	2	[7, 78, 79]
p.T320I	Missense	ULD	ALS	0.626	-0.15	0.14			[6, 18, 78, 79]
p.H322Y	Missense	ULD	ALS	0.982	0.65	0.14	64	2	[19, 78, 79]
p.331sp	Splice	ULD	ALS						[6, 78]
p.T331I	Missense	ULD	ALS	0.971	-0.08	0.00			[6, 78, 79]
p.I334T	Missense	ULD	ALS	0.002	1.94	0.84	51	4	[16]
p.T343S	Missense	ULD	ALS	0.993	-2.13	0.00			[6, 78, 79]
p.R357Q	Missense	ULD	ALS	0.105	0.57	0.03	61	3	[6, 7, 79]
p.R357X	Nonsense	ULD	ALS						[6, 78, 79]
p.R384T	Missense	ULD	ALS	0.986	1.47	0.67		2	[17]
p.Y394D	Missense	ULD-CCD1	ALS	0.940	1.57	0.09			[6, 78, 79]
p.S398PfsX11	Deletion	ULD-CCD1	ALS				59	5	[19, 78, 79]
p.L399fs	Frameshift	ULD-CCD1	ALS				62	4	[6, 14, 78]
p.P400Lfs	Deletion	ULD-CCD1	ALS						[14, 79]
p.A417X	Splice	CCD1	ALS, ALS-FTD				65, 56, 62	7, 2, 6	[7, 78, 79]
P.V421fs	Frameshift	CCD1	ALS						[6, 78]
p.C423Lfs	Deletion	CCD1	ALS						[6, 79]
p.R440Q	Missense	CCD1	ALS	0.997	1.29	0.03			[6, 78, 79]
p.R440X	Nonsense	CCD1	ALS, ALS-FTD, FTD				47, 58, 73	13, 3, 4	[6, 7, 18, 78, 79]
p.R444X	Nonsense	CCD1	ALS				57	2	[6, 15, 78, 79]
p.R444Q	Missense	CCD1	ALS	0.997	0.73	0.28	72	1	[17]
p.I450KfsX15	Deletion	CCD1	ALS, ALS-FTD				57, 71, 77, 71, 51, 55, 54	2, 3, 1, 2, 2, 3, 3	[7, 78, 79]
p.T462fs	Frameshift	CCD1	ALS						[6, 78]
p.E463Sfs	Deletion	CCD1	ALS						[6, 79]
p.C471Y	Missense	CCD1	ALS	0.886	2.08	0.08			[6, 78, 79]
p.V479EfsX4	Deletion	CCD1	ALS-FTD				65, 58	1, 3	[7, 78, 79]
p.Y482X	Nonsense	CCD1	ALS-FTD						[18, 78, 79]
p.S499X	Nonsense	CCD1	ALS						[6, 78, 79]
p.D500fs	Frameshift	CCD1	ALS						[6]
p.I501Kfs	Insertion	CCD1	ALS						[6, 78, 79]
p.I515T	Missense	CCD1	ALS	0.002	0.78	0.48	59	10	[19, 78, 79]
p.S518LfsX32	Insertion	CCD1	ALS				64	1	[19, 78]
p.I522M	Missense	CCD1	ALS	0.929	1.99	0.11			[6, 78, 79]
p.A535T	Missense	CCD1	FTD	0.000	1.71	0.44	52	10	[19, 78, 79]
p.E550fs	Frameshift	CCD1	ALS						[6, 78]
p.L552Tfs	Insertion	CCD1	ALS						[6, 79]
p.M559R	Missense	CCD1	ALS	0.012	-0.51	0.00	60	6	[7, 78, 79]
p.Q565P	Missense	CCD1	ALS	0.990	-1.26	0.01			[6, 78, 79]

Mutation	Type of Variant	Location	Disease	PolyPhen2	SNPs3D	SIFT	Age of onset	Survival Time	Refs
p.A571V	Missense	CCD1	ALS	0.455	-0.39	0.26			[6, 79]
p.R574Sfs	Splice	CCD1	ALS						[6, 79]
p.Q581H	Missense	CCD1	ALS	0.990	-1.71	0.01			[6, 78, 79]
p.587sp	Splice	CCD1	ALS						[6, 78]
p.M598V	Missense	CCD1	ALS	0.001	1.09	0.31			[6, 78, 79]
p.Q629fs	Deletion	CCD1	ALS						[6, 78, 79]
p.E640del	Deletion	CCD1	ALS						[6, 78]
p.E643del	Deletion	CCD1	ALS, ALS-FTD, FTD				69, 62, 62, 63, 41, 63, 64, 70, 69	3, 11, 11, 3, 1, 6, 13, 1, 9, 6, 7	[7, 19, 78, 79]
p.653sp	Splice	CCD1	ALS-FTD						[6, 78]
p.L654Vfs	Splice	CCD1	ALS-FTD						[18, 79]
p.Q655X	Nonsense	CCD1	ALS-FTD						[6, 18, 78, 79]
p.M662T	Missense	CCD2	ALS-FTD	0.004	0.76	0.49			[18, 78, 79]
p.M690fs	Frameshift	CCD2	ALS-FTD					9	[17]
p.690-713del	Splice	CCD2	ALS, ALS-FTD, FTD				63, 52, 74, 65, 52, 64, 78, 65	4, 2, 1, 5, 1, 3, 2	[7, 78, 79]
p.E696K	Missense	CCD2	ALS-FTD, FTD	0.734	0.32	0.04	78	6	[7, 20, 78, 79]
p.I710N	Missense	CCD2	ALS	0.790	-0.75	0.00			[6, 78, 79]

Additional references

78. Ahmad L, Zhang SY, Casanova JL, Sancho-Shimizu V: Human TBK1: A Gatekeeper of Neuroinflammation. Trends in molecular medicine 2016, 22(6):511-527.

79. Freischmidt A, Muller K, Ludolph AC, Weishaupt JH, Andersen PM: Association of Mutations in TBK1 With Sporadic and Familial Amyotrophic Lateral Sclerosis and Frontotemporal Dementia. JAMA neurology 2016.

Prediction tools

PloyPhen2	1 = probably damaging<br>0 = benign<br>middle = possibly damaging
SNPs3D	Non-deleterious = +<br>Deleterious = -<br>Larger the score = more confident
SIFT	Deleterious = <0.05

low

medium

high
